# 153 ”We’re unburdened” - Audio diaries as a novel and inclusive approach to movement behaviours in mental health

**DOI:** 10.1093/eurpub/ckae114.030

**Published:** 2024-09-26

**Authors:** Ilaria Pina, Kirstie Anderson, Emily J Oliver

**Affiliations:** Newcastle University, Newcastle upon Tyne, United Kingdom; Newcastle University, Newcastle upon Tyne, United Kingdom; Newcastle upon Tyne Hospitals NHS Foundation Trust, Newcastle upon Tyne, United Kingdom; Newcastle University, Newcastle upon Tyne, United Kingdom

## Abstract

Physical activity, sedentary time and sleep are traditionally measured with quantitative approaches, limiting the possibility to capture information on type and context of these behaviours. Here, we adopted a flexible approach with audio diaries to explore movement behaviours in people living with severe mental illness (SMI). Audio diaries are an affordable approach, potentially able to overcome health inequalities and literacy levels. However, the use of this method in exploring movement behaviours in mental health is limited.

Qualitative data were generated using audio diaries for 7 days with 9 participants experiencing SMI. Semi-structured interviews on the method were performed at the end of data generation. These were conducted online or in-person and analysed using reflexive thematic analysis. This study was grounded ontologically in relativism and epistemologically in constructionism, with an experiential orientation to data interpretation to capture participants’ perspectives of movement behaviours. The protocol for the use of audio diaries has been co-produced with a lived experience advisory panel.

Four themes were developed: finding themselves in a “vicious circle” with physical, mental issues impacting movement behaviours and vice versa; daily internal dialogue – repetitive thoughts that attempt to override daily functioning, movement and sleep; “Do not let fatigue win” by pushing through the day – motivation vs commitment; positive AND negative awareness of the importance of movement behaviours. The fluidity in speech enabling an immediate response and the control over how participants recorded their account, led to high acceptability and completion rates. The use of audio diaries was experienced as cathartic in that it helped to reflect upon the challenges in movement behaviours when the emotional load had a severe impact on daily life.

Participants showed awareness of the need to be physically active. However, when people faced increased mental or physical burden, this contributed to feelings of frustration, with completion of activities driven by guilt rather than enjoyment. Attention should be given to public health messaging for movement behaviours in mental health with involvement of people with lived experience of SMI. The value of verbalisation with audio diaries could offer an in-depth understanding of processes underpinning health-related behaviours.

